# The effect of frontoparietal paired associative stimulation on decision-making and working memory

**DOI:** 10.1016/j.cortex.2019.03.015

**Published:** 2019-08

**Authors:** Camilla L. Nord, Traian Popa, Emma Smith, Ricci Hannah, Nuria Doñamayor, Kathrin Weidacker, Paul M. Bays, John Rothwell, Valerie Voon

**Affiliations:** a Department of Psychiatry, University of Cambridge, Cambridge, UK; b MRC Cognition and Brain Sciences Unit, University of Cambridge, Cambridge, UK; c Center for Neuroprosthetics, École Polytechnique Fédérale de Lausanne, Switzerland; d National Institute of Neurological Disorders and Stroke, National Institutes of Health, Bethesda, MD, USA; e Sobell Centre for Motor Neuroscience and Movement Disorders, University College London, London, UK; f Department of Psychiatry and Psychotherapy, Charité – Universitätsmedizin Berlin, Germany; g Department of Psychology, University of Cambridge, Cambridge, UK; h Cambridgeshire and Peterborough NHS Foundation Trust, Cambridge, UK

**Keywords:** Paired associative stimulation, Decision-making, Habitual, Goal-directed, Psychiatry

## Abstract

Previous single-site neurostimulation experiments have unsuccessfully attempted to shift decision-making away from habitual control, a fast, inflexible cognitive strategy, towards goal-directed control, a flexible, though computationally expensive strategy. We employed a dual-target neurostimulation approach in 30 healthy participants, using cortico-cortical paired associative stimulation (ccPAS) to target two key nodes: lateral prefrontal cortex (LPFC) and intraparietal sulcus (IPS), to test whether decision-making can be artificially shifted from habitual toward goal-directed control. Participants received three active stimulations, delivered at least six days apart (each involving 100 paired pulses over the IPS and LPFC, varying the interstimulus interval): two interventional, time-relevant ccPAS (10 msec interval) and one control, non-time-relevant ccPAS (100 msec interval). Following stimulation, participants completed a sequential learning task, measuring goal-directed/habitual control, and a working memory task. IPS→LPFC ccPAS (stimulating IPS, then LPFC with a 10 msec interval) shifted decision-making from habitual toward goal-directed control, compared to control ccPAS. There was no effect of LPFC→IPS ccPAS, nor an effect of any PAS condition on working memory. Previous studies have shown ccPAS effects outside the motor domain targeting prefrontal regions on response inhibition, attentional bias, and alpha asymmetry. The present study measures the behavioural effects of parietal-prefrontal PAS, focusing on a highly complex decision-making task and working memory. If confirmed in larger studies, this would be the first instance of neurostimulation successfully shifting decision-making from habitual to goal-directed control, putatively via inducing long-term potentiation between the IPS and LPFC. However, we found no effect in the other direction (LPFC→IPS ccPAS), and no effect on working memory overall. PAS is a relatively new neuromodulatory technique in the cognitive arsenal, and this study could help guide future approaches in healthy and disordered decision-making.

## 1 Introduction

When animals make choices, their decisions are influenced by two modes of control: a fast, but inflexible ‘habitual’ control, and a slower, but flexible ‘goal-directed’ or deliberative control. Mediation between these two strategies is key to adaptive decision-making. These strategies are formalised as two separate but concurrent update rules in a popular computational framework: ‘model-free’ (habitual) and ‘model-based’ (goal-directed) reinforcement learning algorithms (Daw, Gershman, Seymour, Dayan, & Dolan, 2011). We have shown that an imbalance between these two different modes of control is characteristic of compulsive disorders, including binge eating disorder, obsessive-compulsive disorder, and methamphetamine addiction (Voon et al., 2015), a finding substantiated by a large-scale study reporting a highly specific association between deficits in goal-directed control and a dimensional psychiatric phenotype, ‘compulsive behaviour and intrusive thoughts’ (Gillan, Kosinski, Whelan, Phelps, & Daw, 2016). Healthy individuals with greater goal-directed control are also less susceptible to habit acquisition (Gillan, Otto, Phelps, & Daw, 2015), while reliance on habits may constitute a vulnerability factor for impulsive-compulsive behaviour (de Wit et al., 2012). Therefore, improving goal-directed control could prevent the instantiation of habitual, compulsive behaviours.

Goal-directed control relies heavily on intact prefrontal cortex (Smittenaar et al., 2013, Voon et al., 2015) and executive functions (Otto, Gershman, Markman, & Daw, 2013). There is a particularly strong relationship between working memory and model-based strategies: greater working memory capacity is associated with more goal-directed decision-making in both young healthy adults (Eppinger, Walter, Heekeren, & Li, 2013) and patients with Parkinson's disease (Sharp, Foerde, Daw, & Shohamy, 2015); a greater working memory capacity even appears protective against the detrimental effect of stress on goal-directed learning (Otto, Raio, Chiang, Phelps, & Daw, 2013). This suggests that the neural substrates of goal-directed decision-making and working memory may be related (Sharp et al., 2015).

Previously, transcranial magnetic stimulation (TMS) has been shown to shift control away from goal-directed and towards habitual control in a sequential learning task by disrupting right lateral prefrontal cortex (LPFC) activity; TMS of the left LPFC produced a similar shift only in participants with low working memory, again supporting a link between working memory and the balance of goal-directed/habitual control (Smittenaar et al., 2013). However, to date, no form of brain stimulation has been found to improve goal-directed control (Smittenaar, Prichard, FitzGerald, Diedrichsen, & Dolan, 2014).

Brain stimulation has classically been attempted over a single node. However, there is an extensive literature linking both normative and pathological cognitive processing to complex network interactions (Fornito et al., 2015, Haber and Behrens, 2014, Park and Friston, 2013). Therefore, neurostimulation has recently begun to move away from classical single-target approaches, which are less physiologically relevant, given the network of interacting regions involved in cognitive processes. In this study, we attempted to increase goal-directed control using a dual-target neuromodulation intervention: cortico-cortical paired associative stimulation (ccPAS) of the right LPFC and intraparietal sulcus (IPS). This technique involves two TMS pulses delivered at predefined intervals over two interconnected regions, and has been shown to modify the responsiveness of at least one of the targets, purportedly via spike timing-dependent plasticity mechanisms (Rizzo et al., 2008, Stefan et al., 2000). For example, ccPAS of the LPFC and posterior parietal cortex can bidirectionally induce spike timing-dependent plastic changes in the LPFC but not in the parietal target, based on the order of the pulses (Casula, Pellicciari, Picazio, Caltagirone, & Koch, 2016). Recently, in the first demonstration of ccPAS in the cognitive domain, we reported putative cortico-cortical and cortico-subcortical effects of ccPAS of the pre-supplementary motor area and inferior frontal cortex on inhibitory behaviour as a function of age (Kohl et al., 2018). This study was followed by a second report of ccPAS in the cognitive domain, which found effects on attentional bias accompanied by bidirectional changes in frontal interhemispheric connectivity depending on the order of stimulation (Zibman, Daniel, Alyagon, Etkin, & Zangen, 2019), replicating the order-dependent effects observed in motor ccPAS (Veniero, Ponzo, & Koch, 2013). Recently, an innovative ccPAS study using resting-state connectivity measures to acquire individualised parietal and prefrontal stimulation targets found bidirectional effects on spontaneous and task-evoked networks (default mode and task-positive) (Santarnecchi et al., 2018). Compellingly, ccPAS modulated the speed of switching between resting-state and task-based networks, with parieto-prefrontal stimulation increasing activation of medial prefrontal regions, and frontoparietal stimulation increasing activation of posterior medial structures (Santarnecchi et al., 2018).

We targeted the right LPFC and IPS as two structurally connected nodes activated in both decision making and working memory tasks (Chafee and Goldman-Rakic, 2000, Mars et al., 2011). A dual-target intervention enabled us to target this network more comprehensively than a single constituent part. The rationale for targeting this network was twofold: it plays an essential role in the sequential learning task (measuring the balance of goal-directed and habitual control) (Gläscher, Daw, Dayan, & O'Doherty, 2010), and it underpins visuospatial working memory (Koch et al., 2005, Rowe and Passingham, 2001, Sauseng et al., 2005). In the sequential learning task, fronto-parietal networks underpin the ‘state prediction error’ learning signal essential to employing flexible, effortful goal-directed control (in contrast, fast but inflexible habitual control is underpinned by striatal reward prediction errors) (Gläscher et al., 2010). A long literature of imaging and brain stimulation experiments has also revealed that long-range fronto-parietal coherence is associated with difficult visuospatial working memory conditions (Sauseng et al., 2005), with functional interconnectedness between the LPFC and parietal cortex crucial in maintaining spatial information in memory (Koch et al., 2005).

We hypothesised that by modulating the connectivity between the right LPFC and IPS using ccPAS, we might shift the behaviour of our healthy subjects toward a more goal-directed strategy. Considering the electrophysiological (but not behavioural) outcome measures of the previous ccPAS study with parietal and prefrontal targets (Casula et al., 2016), we could expect ccPAS to induce plastic effects in the LPFC. However, the importance of the directionality (prefrontal-parietal or parietal-prefrontal) for any behavioural effect is not yet known; thus, we tested this by employing one ccPAS intervention that we hypothesised would modify prefrontal-parietal connectivity (right LPFC→iPS ccPAS) and one that would modify parieto-prefrontal connectivity (right iPS→LPFC ccPAS), by varying the timing between pulses. Using a well-established task and computational model (Daw et al., 2011), we measured the balance between goal directed and habitual control after each ccPAS intervention. We also measured visuospatial working memory as a key variable due to its crucial role in the balance between goal-directed and habitual control (Eppinger et al., 2013, Otto et al., 2013b, Sharp et al., 2015, Smittenaar et al., 2013).

## 2 Methods and materials

### 2.1 Participants

We recruited 33 individuals from the general population to take part in the study, using emails to a healthy participant database and posters. The study was undertaken according to the Helsinki declaration, with the understanding and written consent of each participant. Three participants dropped out before attending all three sessions: two disliked the stimulation, and one was unable to attend the remaining sessions. The final sample included 30 participants who reported no history of psychiatric or neurological disorder, or any counter-indication for TMS (including, but not limited to, a family history of seizures, implanted electronic devices, and metal in the head or neck). All but one participant were right-handed; we follow up our key analyses with analyses excluding the non-right-handed participant.

### 2.2 Experimental design

Participants underwent three active ccPAS conditions at least six days apart; the order of the three conditions was randomized using a custom-written algorithm. In one condition (LPFC→IPS), the TMS pulse over right LPFC preceded the pulse over right IPS by 10 msec; in the second, the pulse over right IPS preceded the pulse over right LPFC by 10 msec (IPS→LPFC); in the control condition, the two pulses were delivered 100 msec apart (randomised so that half the participants received LPFC→IPS and half IPS→LPFC), with a dedicated analysis confirming no effect or difference between the two directions at this interval. The inter-stimulus intervals were chosen based on previous M1 ccPAS protocols, where 8–10 msec was successfully used for activating oligosynaptic connections of similar length: parietal cortex→M1 (Karabanov, Chao, Paine, & Hallett, 2013); interhemispheric M1→M1 (Rizzo et al., 2008), and LPFC→parietal (Casula et al., 2016). The ccPAS was immediately followed by the behavioural tasks (Bays et al., 2009, Daw et al., 2011) and monetary choice questionnaire (Kirby, Petry, & Bickel, 1999), all falling within the 30 min in which the ccPAS effect is presumed to be significant (Stefan et al., 2000).

We report the effects of ccPAS targeting the right LPFC and right IPS on the two-step reinforcement learning task (Daw et al., 2011) and a visuospatial working memory task (Bays et al., 2009), described below. Participants were trained on both tasks on the first testing day, prior to TMS, and were verbally reminded of the task instructions on each subsequent testing day.

### 2.3 ccPAS set-up and protocol

**Fig. 1:**
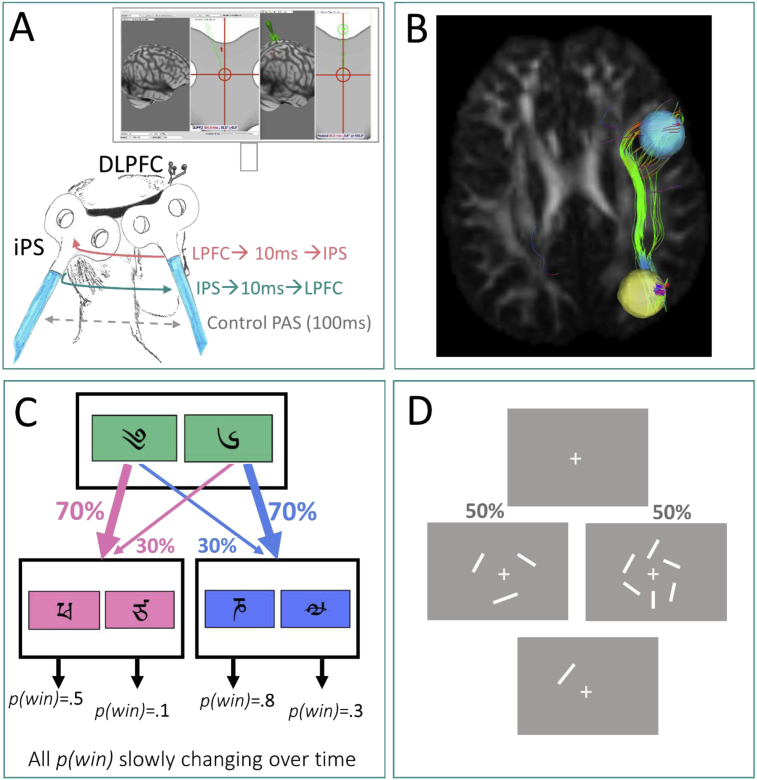
**Neurostimulation montage and task design**. A. Cortico-cortical paired associative stimulation (ccPAS) consisted of one coil positioned over the right intraparietal sulcus (IPS) (20° tilt posteriorly) and one coil positioned over the right lateral prefrontal cortex (LPFC) (20° tilt anteriorly), under neuronavigation (see insert). B. Illustration of target regions [x,y,z, in Montreal Neuroimaging Index (MNI) coordinates (in mm)]: the right LPFC (blue: 50,16,26), and right inferior parietal sulcus (yellow: 30,–60,50). For visualisation purposes only, we depict an example of the white matter tracts connecting IPS and LPFC coordinates. This illustration was made using previously-published diffusion-weighted imaging data from the Human Connectome Project (healthy subject dataset), employing deterministic tractography on a standardized structural connectome [see previous publication for methodological details and full results (Horn et al., 2017)]. C. The two-step task involved a first level of selection between two symbols, with each having a fixed probability of leading to a subsequent set of stimuli. At the second level, participants selected one of the new symbols, which were each associated with a differential probability of monetary reward. The second-level contingencies (i.e., reward probabilities) shifted slowly and independently over time according to Gaussian random walks; one example is illustrated in Fig. 1C). D. The working memory task began with a fixation cross (500 msec) before presenting either three or six lines rotated around the fixation cross (3 lines in 75 trials; 6 lines in 75 trials; randomised order across trials), also for 500 msec. Last, participants had to rotate a probe line using the mouse, attempting to match its orientation to the line displayed in that location on the previous screen (unlimited time allowed).

The ccPAS was delivered with two Magstim machines (Magstim 200^2^ and Magstim BiStim^2^) machines and two 70 mm figure-of-eight coils (The Magstim Company Ltd., United Kingdom) delivering mono-phasic pulses. The coils were positioned (see Fig. 1A) over the posterior part of the right inferior frontal gyrus/BA44 (here referred to as LPFC) and posterior part of the right IPS under neuronavigation (Brainsight; Rogue Research Inc., Montreal, Quebec, Canada). We selected targets that were common across both behavioural tasks; as the field is lacking meta-analyses of goal-directed/habitual control imaging studies, we selected coordinates based on previous meta-analyses of imaging studies of working memory-related tasks (Rottschy et al., 2012) that overlapped with functional activation from an fMRI study of the goal-directed/habitual task we used (Gläscher et al., 2010) [x,y,z, in Montreal Neuroimaging Index (MNI) coordinates (in mm)]: 30,–60,50 and 50,16,26. [Note that targeting these coordinates overlaps with the lateral prefrontal and inferior parietal activation reported in the Gläscher study, given the ≥1 cm area stimulated by TMS (Opitz, Zafar, Bockermann, Rohde, & Paulus, 2014)]. For illustrative purposes only, we include an example of the white matter tractography between the two coordinates we targeted with ccPAS (lateral prefrontal and inferior parietal) (see Fig. 1B); this was computed on existing diffusion-weighted images from the Human Connectome Project [see previous publication for methodological details and results (Horn et al., 2017)].

These coordinates were targeted across all conditions (including control stimulation): coil 1 was positioned over the right LPFC at ∼20° from the coronal plane with the handle pointing anteriorly, and coil 2 over the right IPS, at ∼20° to the coronal place with the handle pointing posteriorly, in order to have the eddy currents induced perpendicular on the respective sulci walls (Fig. 1A). The use of neuronavigation for exact coil placing allowed precise reproduction of the stimulation conditions across sessions on each testing day.

The ccPAS protocol consisted of 100 pairs of pulses delivered at .2 Hz (8.3 min total duration). The intensity of both IPS and LPFC stimuli were set to 120% resting motor threshold (RMT; defined as the minimum stimulation intensity to produce a motor evoked potential >.05 mV in the first dorsal interosseous muscle of the left hand in five out of ten consecutive trials). The RMT was determined under electromyographic monitoring using surface electrodes (in a belly tendon montage) connected to a BioPak amplifier, and visualised in AcqKnowledge Software (BioPak Systems Inc., California, USA).

### 2.4 Behavioural measures

#### 2.4.1 2-Step task

The two-step reinforcement learning task is a well-established decision-making paradigm that measures the influences of two separable systems: model-free (habitual) and model-based (goal-directed) learning (Daw et al., 2011). Briefly, the task consists of two stages. In the first stage, participants selected one of two symbols, which have a fixed differential probability of leading to a second pair of symbols (see Fig. 1C). In the second stage, participants selected one of the new pair of symbols, which have their own differential probability of a monetary reward outcome. The second stage reward probabilities (between each secondary symbol and reward outcome) slowly changed trial-by-trial according to random walks. We randomly assigned one of three random walks to each participant, to ensure effects were not dependent on a specific reward structure.

A participant's first-level choices are influenced by two factors: whether the previous trial was rewarded (model-free), and whether the reward or its absence resulted from a common or rare transition between first-level and second-level stimuli (model-based). The degree to which each factor influences behaviour varies between participants, and is quantifiable using a modified reinforcement learning model (described below in the Task analysis section) (Daw et al., 2011).

There were 67 trials in the task. Each trial consisted of the first-level stimuli (displayed for 2.22 sec), the second level stimuli (also displayed for 2.22 sec), and the outcome display of a pound coin or a circle with a cross through it (shown for 1.11 sec), representing a win or no win, respectively. If the participant did not respond in the 2.22-sec response period for either stimuli pair, the trial was skipped and the next trial began. The inter-trial interval was jittered between 0 and 3.6 sec, inclusive (mean jitter: 1.8 sec). The inter-stimulus interval was .8 sec. The task lasted approximately 7 min.

#### 2.4.2 Working memory task

The visuospatial working memory task we employed was an orientation delayed-estimation task, a variant of a previously-described paradigm (Bays et al., 2009). Each trial began with a central fixation cross (white, on a grey screen, presented for 500 msec), after which participants were briefly presented with either three or six white lines of varying orientations. The lines were presented in an invisible circle around a central dot (presented for 500 msec), after which the lines disappeared and were replaced with one ‘probe’ line in the spatial location of one of the previous lines (see Fig. 1D). Participants were instructed to match the orientation of the probe line with the orientation of the target line appearing in the same location (using a computer mouse to rotate the line; no maximum time to respond). There was a 1s delay after responding before the next trial began. Participants completed 150 trials: 75 in the low-load working memory condition (3 lines) and 75 in the high-load condition (6 lines). Trials were presented intermixed in a randomised order, with an optional rest offered every 25 trials. The task lasted between 9 and 11 min.

#### 2.4.3 Questionnaires

On the first day of testing, participants completed three clinical questionnaires: the Obsessive-Compulsive Inventory (Revised) (OCI-R) (Foa et al., 2002); the Beck Depression Inventory (BDI) (Beck, Steer, & Brown, 1996, pp. 78204–82498); and the State-Trait Anxiety Inventory (STAI) (Spielberger, Gorsuch, & Lushene, 1970).

### 2.5 Analysis

#### 2.5.1 2-Step task: reinforcement learning model analysis

We fit behavioural data from the 2-step task to a hybrid learning algorithm designed for this task (Daw et al., 2011). This model has been extensively validated with computer simulations and participant data for use in the two-stage task (Daw et al., 2011, Wunderlich et al., 2012). Our model consisted of the following parameters: a choice reliability parameter (β) for stages 1 and 2 (limits: 0–∞), learning rate (α) for stages 1 and 2 (limits 0–1), a reinforcement eligibility parameter (λ) (limits 0–1), perseveration rate (limits –∞-∞), and a weighting parameter (*w)* (limits 0–1). For the full model, please see Appendix A.

Our analyses were constrained to the *w* parameter – our key outcome variable and a measure of goal-directed (model-based) and habitual (model-free) control. Essentially, *w* ranges from 0 to 1, and can be thought of as the relative influence of model-free and model-based systems. For a given participant, if *w* is less than .5, they are more reliant on the habitual, model-free system; if it is greater than .5, they are more reliant on the model-based, goal-directed system; a value of .5 would indicate a balanced influence of both systems on behaviour.

#### 2.5.2 Visuospatial working memory task analysis

For the working memory task, we calculated a measure of error (the angular deviation between the subject's reported orientation and the original target orientation), and from this, a measure of recall precision (the reciprocal of the SD of error in response), which we considered our outcome variable on this task (Bays et al., 2009).

#### 2.5.3 Statistical approach

In each analysis, we first verified using between-subjects tests that our two 100 msec conditions (100 msec LPFC preceding IPS and 100 msec IPS preceding LPFC, randomly allocated to participants) did not differ from one another; after verifying this, we merged the data sets, considering them as a single control condition.

In all analyses, we first attempted to perform parametric statistics: we assessed the normality of raw data using Kolmogorov-Smirnov tests, transforming the data if it was non-normal using common transformations. Next, we assessed the normality of the transformed data again using Kolmogorov-Smirnov tests, performing parametric statistics if any transformation normalised the data. Only if the data remained non-normally distributed after transformation did we perform non-parametric statistics.

### 2.6 Power calculation

We calculated that we would need 29 participants to detect a medium-to-large effect size (Cohen, 1988) of d_z_ = .7 (two-tailed matched pairs *t*-test, alpha = .05) with 95% power [calculated in G*Power 3.1.9.2 (Faul, Erdfelder, Lang, & Buchner, 2007)] on either task. Note, however, that this may have been insufficient to detect smaller effect sizes; we have previously shown a larger sample size is required for sufficient power to detect subtle (e.g., r = .3) relationships with questionnaire measures (Nord, Prabhu, et al., 2017). However, given both the exploratory nature of our novel neuromodulation study and our eventual goal to detect effect sizes of clinical significance, we powered this study to detect a medium-to-large effect size.

### 2.7 Data confirmation statement

We confirm that we have reported how the sample size was determined (power calculation), all data excluded (see 3.1 for detail, one participant's data was not analysed), our inclusion and exclusion criteria (no history of brain disorder; no counter-indications for TMS), that our inclusion/exclusion criteria was applied prior to data analysis, all manipulations (three TMS conditions) and all measures (2-step task, working memory task, and questionnaires).

## 3 Results

### 3.1 Participants

Thirty participants completed all three testing sessions (mean age = 35.90, SD = 14.40; 18 females). For all participants, we verified that the margin of error of the neuronavigation targeting was under 5 mm for each target coordinate before and after stimulation to ensure adequate targeting of ccPAS. One participant's data was not analysed due to their neuronavigation marker slipping partway through ccPAS stimulation, resulting in inaccurate targeting for part of the stimulation session.

### 3.2 2-Step task: effect of ccPAS on computational parameters

We found no difference between the two 100 msec control conditions in either task, so we collapsed across both for a single control condition (see Appendix B for full statistics). In our initial model, we included age, gender, and order (coded as day participants received control stimulation). As there was no effect of age or gender (both *p* > .4), we removed these from the model; we retained order in the model as there was a significant interaction between order and the effect of ccPAS condition [F(4,52) = 3.05, *p* = .025]. The interaction between order and ccPAS condition was highly complex. Specifically, participants who received control stimulation on day 1 or day 2 (N = 21) had higher *w* following IPS→LPFC PAS (received on day 2 or 3) than following the other two conditions; participants who received control stimulation on day 3 (N = 7) had slightly lower *w* following IPS→LPFC ccPAS than *w* following LPFC→IPS ccPAS. See Appendix C for Table C.1 presenting all six order combinations and their associated performances across conditions.

**Fig. 2:**
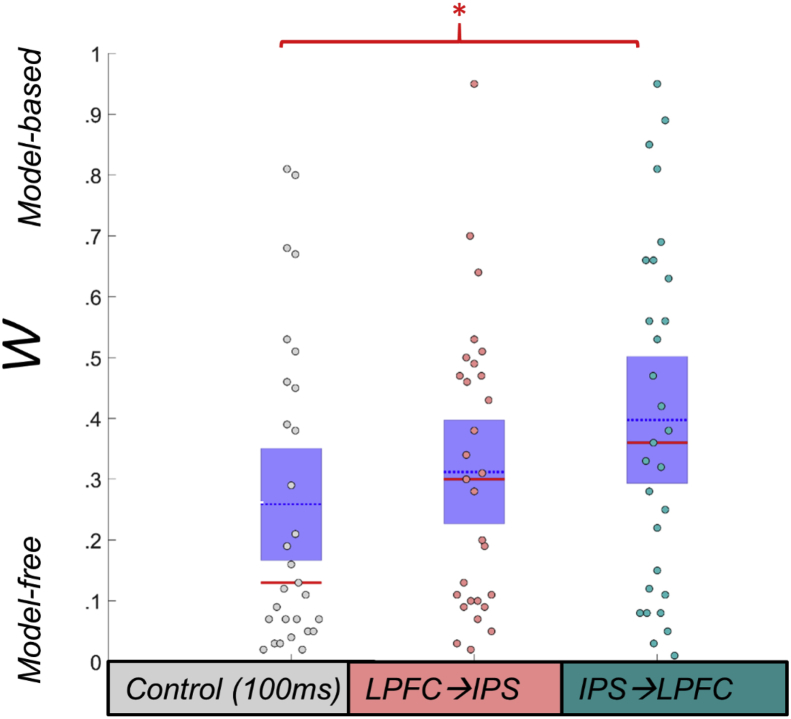
**Two-step task performance under ccPAS conditions.** The weighting of goal-directed (model-based: closer to w = 1) to habitual control (model-free: closer to w = 0) following control stimulation (grey points), lateral prefrontal cortex (LPFC) → intraparietal sulcus (IPS) 10 msec interval ccPAS (pink points), and following IPS→LPFC 10 msec interval ccPAS (teal points). In the IPS→LPFC condition, participants significantly shifted toward model-based (goal-directed) and away from model-free (habitual) control of behaviour, compared to the control condition (non-parametric test, *p* = .028). Red line = median; blue dotted line = mean; purple patch = standard error of the mean; * = *p* < .05.

There was a significant main effect of ccPAS condition in a repeated-measures analysis of variance: F(2,52) = 4.62, *p* = .014 (see Fig. 2). This effect was driven by the difference between IPS→LPFC and the control condition [paired contrast: t(28) = 2.58, *p* = .015] and had a medium effect size [(Cohen, 1988)*r* = .284]. There was no difference between LPFC→IPS and the control condition [t(28) = .879, *p* = .387]. Excluding the one non-right-handed participant did not change these results substantially: F(2,50) = 4.97, *p* = .011 for the effect of ccPAS condition and t(270 = 2.56, *p* = .016) for the paired contrast between IPS→LPFC and the control condition. The difference between IPS→LPFC and the control condition survived correction for the two linear contrasts (corrected alpha = .025).

However, for the control condition, *w* was highly positively skewed, and significantly non-normally distributed (Kolmogorov-Smirnov statistic *p* = .003; skew = .954 (SE = .434); see Fig. 2 for individual data points), and no transformation of the data (including log, arcsine, etc.) normalised the distribution. Therefore, we replicated our parametric analyses with a non-parametric ranked analysis of covariance, which was not significant (or marginally so): F(2,86) = 2.38, *p* = .098, and replicated our parametric linear contrast with a related-sample Wilcoxon signed-rank test between IPS→LPFC and the control condition (significant, *p* = .028, again indicating a shift towards goal-directed control following IPS→LPFC ccPAS). As with the parametric analyses, excluding the one non-right-handed participant did not change results from our non-parametric analyses substantially, either for the ranked analysis of covariance testing the effect of ccPAS condition [F(2,83) = 2.42, *p* = .095], nor for the Wilcoxon signed-rank test between IPS→LPFC and the control condition (*p* = .031).

Intraclass correlation coefficient analysis (2-way mixed: assesses the average reliability of individuals' performance, measuring consistency, rather than absolute values) showed very good within-subject reliability across the three testing sessions (ICC = .560, *p* = .004), indicating (a) that the 2-step task was a reliable measure; and (b) that, in general, more goal-directed individuals (relative to the rest of the group) after control stimulation remained more goal-directed (relative to the rest of the group) following both stimulation types; that is, the intervention did not alter the overall pattern of the group.

### 3.3 2-Step task: effect of ccPAS on reaction time analysis

We analysed second-stage reaction times (which were normally distributed) by their transition type (rare or common). Here, more goal-directed participants would show slowing on second-stage trials following rare transitions compared to common transitions. Again, we initially included age, gender, and order (day of control stimulation) in the model, but in this case none were significant (all *p* > .1). In the final model, we found a significant effect of transition type on reaction times [F(1,28) = 12.54, *p* = .001; no main effect of ccPAS: F(2,56) = 1.30, *p* = .281; and no interaction effect (possibly a marginal effect) between the two F(2,56) = 2.43, *p* = .097]. This marginal interaction was such that rare transitions slowed participants most following IPS→LPFC stimulation (interpreted as more goal-directed), intermediately with control stimulation, and least after LPFC→IPS stimulation [linear contrast between the two active ccPAS conditions: t(28) = 1.91, *p* = .067; all other contrasts *p* > .1]. None were significant at our corrected threshold (here, with three multiple comparisons: *p* = .0167). This remained true after exclusion of the non-right-handed participant [t(27) = 2.07, *p* = .048].

### 3.4 Working memory: effect of ccPAS on precision

For the visuospatial working memory task, the raw data distribution of our key measure, working memory precision, was also non-Gaussian but in this case was normally distributed following natural log transformation. The model included the independent factors memory load (i.e., the two working memory conditions) and stimulation (i.e., the three ccPAS conditions), in a 2-by-3 repeated-measures ANOVA design. We initially included age, gender, and order (day of control stimulation) as covariates; neither age nor gender interacted significantly with a variable of interest (*p* > .1) and were subsequently removed from the model. However, there was an interaction between order and ccPAS condition on working memory precision [F(4,50) = 3.19], *p* = .021; therefore, this covariate was retained in the model.

**Fig. 3:**
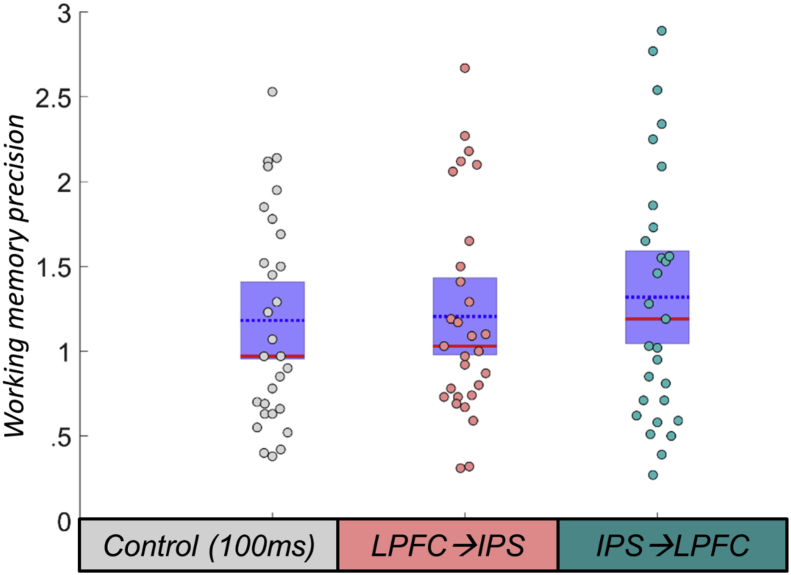
**Working memory performance under ccPAS conditions.** Effect of ccPAS on precision of responses (inverse of the SD), our key measure on the visual working memory task, under control stimulation (grey points), lateral prefrontal cortex (LPFC) → intraparietal sulcus (IPS) stimulation (pink points), and following IPS→LPFC stimulation (teal points). There was no main effect of ccPAS condition on working memory (*p* = .369). Red line = median; blue dotted line = mean; purple patch = standard error of the mean.

In the final model, precision was substantially lower in the high-load working memory condition [F(1,25) = 338.35, *p* < .001]; there was no interaction between memory load and stimulation on working memory precision [F(2,50) = .007, *p* = .993], nor was there a main effect of ccPAS condition on working memory precision [F(2,50) = 1.02, *p* = .369] (excluding the non-right-handed participant did not change the results: both *p* > .5) (see Fig. 3).

Measures of average precision also showed excellent reliability across all three ccPAS conditions (ICC = .956, *p* < .001) (the same was true for raw scores including high and low working memory conditions for each ccPAS condition: ICC = .950, *p* < .001).

### 3.5 Relationship between shift in goal-directed control and working memory

We next assessed whether the reported shift in *w* (towards goal-directed control) following IPS→LPFC ccPAS was associated with either baseline working memory (working memory precision after control stimulation), or the increase in working memory following IPS→LPFC ccPAS. To assess the latter, we calculated a ratio: for *w* (Δ*w* = *w*_control_/*w*_IPS→LPFC_); for working memory precision (Δp = *p*_IPS→LPFC_*/p*_control_).

There was no association between baseline working memory precision and Δ*w* (r = −.087, *p* = .655), nor between Δ*w* and Δp (r = −.052, *p* = .788) (excluding the non-right-handed participant, both *p* > .5).

We verified the lack of association between Δ*w* and Δp using a Bayesian correlation analysis, which showed evidence for the null hypothesis: logBF10 = −1.05 [using the interpretation of Jeffreys (Jeffreys, 1961), as described in detail previously (Nord, Forster, et al., 2017)].

### 3.6 Relationship with self-report clinical measures

We did not find a relationship between any baseline clinical measure (OCI-R, BDI, monetary choice questionnaire, and STAI) and Δ*w* following IPS→LPFC stimulation, or with Δp following IPS→LPFC stimulation: all *p* > .1.

## 4 Discussion

In this study, we investigated the effect of ccPAS of the right IPS and right LPFC on goal-directed versus habitual control, and visuospatial working memory. Compared to control stimulation, we found that ccPAS timed to stimulate the IPS first followed by the LPFC shifted control towards a goal-directed strategy, but this effect was preliminary and, notably, the distribution under control ccPAS was highly skewed. We did not find a relationship between increased goal-directed control and working memory precision, nor an overall effect on working memory precision, implying that the possible plastic change induced by the ccPAS in the LPFC might differently affect goal-directed control and working memory processes.

We have previously shown that ccPAS, usually applied to the motor circuits, can also alter certain behavioural outcomes (Kohl et al., 2018). Critically, we did not demonstrate a main effect of ccPAS in our previous findings but rather an alteration of response inhibition as a function of age. Insofar as we are aware, our preliminary result here is the first demonstration that at least one brain stimulation intervention can increase goal-directed control. Previous attempts used single-site 5 Hz theta burst stimulation [increasing habitual control (Smittenaar et al., 2013), likely due to a disruption of the complex mechanisms responsible for model-based control] or anodal transcranial direct current stimulation (finding no effect) (Smittenaar et al., 2014). In contrast, pharmacological modulation (most notably dopamine supplementation) has been found to increase goal-directed control in both healthy populations (Wunderlich et al., 2012) and patients with Parkinson's disease (Sharp et al., 2015). In the latter study, restoration of goal-directed control was interpreted as the effects of the amino acid precursor to dopamine, levodopa, on the prefrontal cortex. We have built on this previous work to propose a noninvasive brain stimulation intervention that appears to modulate goal-directed behaviour by altering IPS-to-LPFC connectivity.

While subcortical regions implicated in compulsivity [for example, the striatum (Ersche et al., 2011)] are not easily accessible to noninvasive brain stimulation, the IPS and LPFC [which also show abnormalities in compulsive disorders (Barrós-Loscertales et al., 2011, Monterosso et al., 2007)] represent realistic anatomical targets. We hoped to develop an intervention that might improve goal-directed control in light of recent research showing diminished goal-directed control across disorders involving compulsive behaviour and intrusive thought (Gillan et al., 2016, Voon et al., 2015). This would add a putative option to existing single-site neuromodulation approaches for disorders of compulsivity, which show some promise, particularly in treating symptoms of obsessive-compulsive disorder (Zhou, Wang, Wang, Li, & Kuang, 2017). We were not fully successful in this aim: we found preliminary support that participants shifted from a more habitual strategy (under control stimulation) towards goal-directed control after potentiating the IPS-to-LPFC projection using ccPAS. However, we found no effect in the opposite direction. Our central finding that PAS_IPS→LPFC_ increases goal-directed control may suggest that a larger portion of the IPS-to-LPFC pathways are involved in decision-making than in working memory, so that they could be influenced by a less specific, group-defined stimulation targets, or, possibly, that the frontoparietal processes underpinning these two processes are at least partly independent.

According to the principles of Hebbian associative learning, long-term potentiation results when a weak input resulting in a postsynaptic potential precedes the postsynaptic action potential from a strong input. Our findings suggest enhancing goal-directed control may be associated with a specific direction of effect, namely IPS preceding LPFC. We did not show an effect in the opposite direction when aiming to potentiate the probable LPFC-to-IPS projection. This contrasts with a TMS-EEG study in which PFC-to-IPS ccPAS enhanced oscillatory activity in the LPFC and IPS-to-LPFC decreased activity in LPFC, leading the authors to suggest that one should expect that IPS-to-LPFC ccPAS should be associated with an inhibitory effect on prefrontal activity (Casula et al., 2016) (this study focused only on EEG outcomes, without concurrent cognitive measures). These findings converge with those from a recent study investigating resting-state and task-based functional connectivity as a result of paired-pulse stimulation: although neither study includes behavioural measures *per se*, both strongly suggest that paired pulse stimulation induces timing-dependent changes in functional dynamics (Santarnecchi et al., 2018). To resolve this contradiction, further studies combining measures of goal-directed control with measures of oscillatory activity and/or functional connectivity are needed.

Speculatively, our effect on goal-directed control may arise due to modulation of the ‘state prediction error’, which appears to be coded in the right IPS and LPFC (Gläscher et al., 2010). ‘State prediction error’ involves the comparison of the values assigned to actual and expected states relevant to model-based goal-directed control whereas ‘reward prediction error’ involves the comparison of values assigned to actual and expected outcomes relevant to model-free habit control. In previous work, the state prediction error in the right IPS was predictive of subsequent choice whereas the LPFC was not, supporting our findings. Potentially, this signal may originate in the right IPS and be propagated to the LPFC, a route PAS_IPS→LPFC_ may have exploited. Repetition of the pulse pairs, with this particular temporal order, could therefore temporarily potentiate the strength of the parieto-prefrontal projections hence enhancing the representation of ‘state prediction error’.

### 4.1 Limitations and future directions

There are several important caveats to our claims. The novelty of this approach [with different ccPAS only having been used in the cognitive domain twice before (Kohl et al., 2018, Zibman et al., 2019)] requires replication in larger studies, as well as specific investigations of its neural basis using other techniques. In addition, any effects we report (both null and positive) require confirmation in a clinically-relevant group, as it is unclear if our results will generalise to populations with disordered decision-making.

Ideally, task-evoked peak coordinates would be used to localise stimulation sites separately for each individual. A recent paired-pulse study demonstrated that targeting group-level fMRI coordinates (for instance those derived from previous research, as in our approach) would not be appropriate in a large subset of participants, due to individual differences in functional connectivity; this elegantly demonstrates the value of defining TMS targets based on individual functional connectivity patterns (Santarnecchi et al., 2018). Previous work has shown that statistical power increases substantially with increasing specificity of localisation: using individual functional MRI scans to localise coil position, an effect of TMS (here, of parietal TMS during a Stroop-like task) was apparent in a sample size of only six subjects, fewer than half of those required to detect an effect when localising with structural MRI or EEG caps (Sack et al., 2009). Therefore, our group-level approach may have contributed to our absence of LPFC→IPS effect on decision-making, and indeed may have also resulted in an absence of effect on working memory altogether. Therefore, individualised fMRI localisation should be employed in the future to clarify accurate estimates of the effect of ccPAS on both goal-directed control and working memory.

Even more importantly, from our data, it is impossible to know whether our control condition (100 msec intervals) was a ‘true’ control: our design rested on an 100 msec interval as implausible for typical ccPAS physiological effects, but these effects are understood best for short-term synaptic behaviour (as those explored by TMS-evoked EEG or EMG potentials), and therefore the interval timing of more complex cognitive processing is as-yet unknown. While the obvious interpretation of our data is a (subtle) shift towards goal-directed control following IPS-to-LPFC ccPAS, an alternative interpretation is that our control condition shifted behaviour towards habitual control through complex, potentially polysynaptic mechanisms. Certainly, participants' data was highly skewed towards habitual control under this condition, and more normally distributed under both ‘active’ PAS conditions. An investigation of the timing of ccPAS (and thus establishing a robust control condition) is essential for future research.

It is possible that our reported effect on goal-directed control is underpinned by dopaminergic mechanisms: this pattern mirrors the effects of levodopa on goal-directed behaviour (Sharp et al., 2015), and the finding that TMS over frontal regions can alter striatal dopamine release (Strafella, Paus, Fraraccio, & Dagher, 2003). Two studies have previously investigated the effects of parietal/prefrontal ccPAS: the first, using EEG, but without cognitive assessments (Casula et al., 2016); the second, using network analysis of resting-state and task-based fMRI (Santarnecchi et al., 2018). The electrophysiological results from the first study (parietal-to-prefrontal ccPAS decreased frontal excitability, while prefrontal-to-parietal ccPAS increased frontal excitability, albeit with slightly different target sites), might predict behaviour counter to our finding that PAS_IPS→LPFC_ increases goal-directed control; one might have predicted parietal-to-prefrontal ccPAS would make participants more habitual. The recent fMRI-guided paired pulse study (Santarnecchi et al., 2018) found parieto-prefrontal stimulation increased prefrontal activation post-stimulation (which may more closely mirror our findings), while prefronto-parietal stimulation increased parietal activation post-stimulation (Santarnecchi et al., 2018). Particularly relevant for our own findings (which were unidirectional, unlike both of these previous studies) is the report that parieto-frontal ccPAS was ultimately more successful in eliciting changes in the interplay between the two networks, default mode and task-positive. The authors speculated that ccPAS delivered at rest may make the default mode target (in the parietal cortex) more responsive than the task-positive target (in the prefrontal cortex) (Santarnecchi et al., 2018). This could explain the directionality of our results.

It is also worth mentioning that both these previous studies using parieto/prefrontal ccPAS, albeit complementary in approach, differ from ours in a fundamental way: they looked at physiological outcomes (i.e., changes in TMS-evoked potentials on EEG, and inter-target functional connectivity defined by the BOLD signal, respectively) (Casula et al., 2016, Santarnecchi et al., 2018), whereas we explored two different, complex, behavioural outcomes (i.e., decision-making and working memory, with different dynamics in the supporting networks and unknown relations to either the TMS-evoked potential or regional functional connectivity). The definition of the targets were also fundamentally different: the fMRI study used individualized targeting derived from a task with low-cognitive load specifically avoiding the engagement of memory processes, while we used group-level targeting, with coordinates derived from studies seeking explicit engagement of memory and decision-making networks.

Moreover, ccPAS effects seem to decay relatively quickly, with no significant changes in fMRI connectivity patterns observed at 40 min (Santarnecchi et al., 2018). Since our working memory task was always performed after the decision-making task (i.e., after ∼15 min from the end of the stimulation) and lasted 10 min (i.e., until ∼25 min after the end of the ccPAS), it is possible that an efficient plastic effect had worn off by that time, thus explaining our significant findings only in decision-making.

Notably, no previous studies have tested the effects of single-pulse parietal stimulation over the IPS on goal-directed control. If our effects were driven solely by this phenomenon, we would expect identical performance across sessions. However, there may be a more complex relationship between parietal and prefrontal stimulation and its timing, which should undoubtedly be explored in future work, using different frequencies.

## 5 Conclusion

Direct targeting of neural abnormalities (and their behavioural correlates) is challenging with typical neuropsychiatric interventions, whether somatic or psychotherapeutic. To this end, brain stimulation (both invasive and noninvasive) has a unique capacity to directly and specifically modulate neural networks, making it an intervention of particular potential utility in neuropsychiatry. Here, we targeted goal-directed control as a dimensional construct underlying compulsivity (Voon, Reiter, Sebold, & Groman, 2017).

We report a preliminary finding that potentiating IPS-to-LPFC connectivity using ccPAS increased goal-directed control, but we did not find an effect in the reverse direction (LPFC-to-IPS), nor did either ccPAS intervention affect working memory, despite our efforts to include both a highly taxing (five distractors) and less taxing (two distractors) working memory load condition. Therefore, this result should be interpreted with caution and understood in the context of key limitations (lack of neural mechanistic measure; lack of individualised targeting based on functional activation). This study could be used to encourage and inform future ccPAS experiments targeting these regions to establish an optimal targeting approach and stimulation parameters before translation into clinical application.

## Preregistration statement

No part of the study procedures or analyses was pre-registered prior to the research being conducted.

## Funding

This work was supported by a Medical Research Council Senior Clinical Fellowship [grant number MR/P008747/1 to VV] and the NIHR Cambridge Biomedical Research Centre; the German Research Foundation [grant number DO1915/1-1 to ND]; and a Senior Research Fellowship from the Wellcome Trust [grant number 106926 to PMB].

## Disclosures

The authors report no biomedical financial interests or conflict of interest.

## Open Practices

The study in this article earned Open Materials and Open Data badges for transparent practices. Materials and data for the study are available at https://osf.io/e2a6h/.
