# Supplementary material for: The effect of frontoparietal paired associative stimulation on decision-making and working memory
Source: Cortex. 2019 Aug;117:266–76. doi: 10.1016/j.cortex.2019.03.015 (PMC6664322; doi:10.1016/j.cortex.2019.03.015)
Supplement: Multimedia component 1 [file mmc1.docx]

**Appendix**

1. *2-step model*

### We have previously described the model applied to the 2-step task (Voon *et al*, 2015). Briefly, the model is a hybrid of a model-free temporal difference (TD) algorithm, describing habitual control, and a model-based reinforcement learning algorithm, describing goal-directed control (based on those applied to this task in a number of previous studies (Daw *et al*, 2011; Gläscher *et al*, 2010; Wunderlich *et al*, 2012)).

### In the model-free algorithm, choices are based on the predicted long-run value (Q_TD_) of each action (a) at each state (s); the TD reward prediction error (δ) updates the model. Each task trial (t) includes a first-stage state (s_1,t_), a chosen action (a_1,t_) leading to a second-state state (s_2,t_) and action (a_2,t_), which is followed by a reward (r_2,t_, either £1 or £0). There are two TD reward prediction errors, the first when the second-stage state is revealed; the second when the reward is revealed; both update the value (Q_TD_) of the preceding state (s_i,t_) and action (a_i,t_).

### *Eq. A.1*

### *Q_TD_(s_i,t_,a_i,t_) = Q_TD_(s_i,t_,a_i,t_)+ a_i_δ_i,t_*

Where the prediction error is represented as:

*Eq. A.2*

***δ_i,t_ = r_i,t_ + Q_TD_(s_i+1,t_,a_i+1,t_) - Q_TD_(s_i,t_,a_i,t_)***

The first update is according to the value of the second-stage state (i.e., no reward is received yet); the second update includes the reward *(r_2,t_)*; each update includes a learning rate parameter *(α_1_, α_2_)*.

The update to the first stage is also influenced by the stage-2 prediction error (i.e. obtained reward). The influence of this is determined by the eligibility trace parameter *λ*:

*Eq. A.3*

### *Q_TD_(s_1,t1_,a_1,t_) = Q_TD_(s_1,t1_,a_1,t_)+ a_1_λδ_2,t_*

The model-based (goal-directed) reinforcement learning algorithm, by contrast, calculate the first-stage action value based on the probability of each action *a_j_* (*j*=*A*, *B*) (i.e. the symbol selected) leading to each second-stage state (*P*(*s_B_*|*s_A_*,*a_A_*)=0.7; (*P*(*s_B_*|*s_A_*,*a_B_*)=0.3; and conversely for *s_C_*):

*Eq. A.4*

***Q_MB_(s_A_,a_j_) = P(s_B_|s_A,_a_j_)maxQ_TD_(s_B,_a_k_) + P(s_C_|s_A,_a_j_)maxQ_TD_(sc_,_a_k_)***

For each first-stage action, a net action value was also calculated, auch that the parameter *w* describes the weighting parameter (i.e., our primary variable of interest in our 2-step task analyses):

*Eq. A.5*

***Q_net_(s_A_,a_j_) = wQ_MB_(s_A_,a_j_) + (1-w)Q_TD_(s_A_,a_j_)***

At both the first and second stage, the softmax equation was used to calculate the probability of a choice, where at each stage (first and second), β*_i_* is an index of choice reliability (inverse temperature); p represents first-stage choice perseveration; and rep(a) simply indicates if a is a first- (=1) or second-stage action (=0):

*Eq. A.6*

***P(a_i,t_ = a|s_i,t_) α exp(β_i_[Q_net_(s_i,t_, a) + p * rep(a)])***

1. *Verification of control condition*

We verified that there were no differences between the directionality of our control conditions (100ms between right IPS and LPFC stimulation, direction of stimulation randomised across participants). In the two-step task, there was no difference in *w* between the two control conditions (independent-samples Kruskal-Wallis test, p=0.539). In the working memory task, there was no difference in precision between the two control conditions for either low or high working memory loads (low: t(27)=1.62, p=0.116); high: t(27)=1.53, p=0.129).

1. *Testing the effect of order*

We observed a highly complex effect of order (i.e. day of control stimulation), as described in the main text. Therefore, we conducted a follow-up analysis will all six possible order combinations included as a between-subjects variable. In this analysis, albeit not powered enough to warrant clear-cut conclusions, there was no interaction between order and ccPAS condition (marginal effect: F(10,46)=1.78, p=0.091). Results are presented below.

| **Order** | **Control ccPAS**  **mean (SD)** | **LPFC🡪IPS ccPAS mean (SD)** | **IPS🡪LPFC ccPAS mean (SD)** |
| --- | --- | --- | --- |
| **1**  *N=9* | 0.256 (0.226) | 0.220 (0.138) | 0.359 (0.242) |
| **2**  *N=6* | 0.540 (0.285) | 0.356 (0.245) | 0.557 (0.281) |
| **3**  *N=5* | 0.211 (0.236) | 0.543 (0.242) | 0.464 (0.307) |
| **4**  *N=2* | 0.112 (0.110) | 0.045 (0.0348) | 0.116 (0.055) |
| **5**  *N=4* | 0.0645 (0.033) | 0.2755 (0.204) | 0.504 (0.350) |
| **6**  *N=3* | 0.144 (0.082) | 0.3408 (0.328) | 0.1336 (0.195) |

**Table C.1** Means and standard deviations for each ccPAS condition, subdivided by order of stimulation sequence. 1 = control, LPFC🡪IPS, IPS🡪LPFC; 2=control, IPS🡪LPFC, LPFC🡪IPS; 3= LPFC🡪IPS, IPS🡪LPFC, control; 4= LPFC🡪IPS, control, IPS🡪LPFC; 5= IPS🡪LPFC, control, LPFC🡪IPS; 6= IPS🡪LPFC, LPFC🡪IPS, control (Order number= day 1, day 2, day 3).

**Appendix references**

Daw ND, Gershman SJ, Seymour B, Dayan P, Dolan RJ (2011). Model-based influences on humans’ choices and striatal prediction errors. *Neuron* **69**: 1204–1215.

Gläscher J, Daw N, Dayan P, O’Doherty JP (2010). States versus rewards: dissociable neural prediction error signals underlying model-based and model-free reinforcement learning. *Neuron* **66**: 585–595.

Voon V, Derbyshire K, Rück C, Irvine MA, Worbe Y, Enander J, *et al* (2015). Disorders of compulsivity: a common bias towards learning habits. *Molecular psychiatry* **20**: 345–352.

Wunderlich K, Smittenaar P, Dolan RJ (2012). Dopamine enhances model-based over model-free choice behavior. *Neuron* **75**: 418–424.
